# Latitudinal trends in the structure, similarity and beta diversity of plant communities invaded by *Alternanthera philoxeroides* in heterogeneous habitats

**DOI:** 10.3389/fpls.2022.1021337

**Published:** 2022-10-06

**Authors:** Hao Wu, Sijin Dong, Benqiang Rao

**Affiliations:** ^1^ College of Life Sciences, Xinyang Normal University, Xinyang, China; ^2^ Wuhan Botanical Garden, Chinese Academy of Sciences, Wuhan, China

**Keywords:** plant invasions, beta diversity, community similarity, latitude, *Alternanthera philoxeroides*

## Abstract

Variations in latitudinal gradients could lead to changes in the performance and ecological effects of invasive plants and thus may affect the species composition, distribution and interspecific substitution of native plant communities. However, variations in structure, similarity and beta (β) diversity within invaded communities across latitudinal gradients in heterogeneous habitats remain unclear. In this study, we conducted a two-year field survey along 21°N to 37°N in China, to examine the differential effects of the amphibious invasive plant *Alternanthera philoxeroides* on native plant communities in terrestrial and aquatic habitats. We compared the differences in the invasion importance value (*IV*), species distribution, community similarity (Jaccard index and Sorenson index) and β diversity (Bray−Curtis index and β_sim_ index) between terrestrial and aquatic communities invaded by *A*. *philoxeroides*, as well as analyzed their latitudinal trends. We found that the *IV* of *A*. *philoxeroides* and β diversity in aquatic habitats were all significantly higher than that of terrestrial, while the terrestrial habitat had a higher community similarity values. The aquatic *A*. *philoxeroides IV* increased with increasing latitude, while the terrestrial *IV* had no significant latitudinal trend. With increasing latitude, the component proportion of cold- and drought-tolerant species in the terrestrial communities increased, and the dominant accompanying species in the aquatic communities gradually changed from hygrophytes and floating plants to emerged and submerged plants. In addition, the aquatic communities had lower community similarity values and higher β diversity in higher latitudinal regions, while terrestrial communities had the opposite parameters in these regions. Our study indicates that the bioresistance capacities of the native communities to invasive *A*. *philoxeroides* in heterogeneous habitats are different; *A*. *philoxeroides* invasion leads to higher community homogenization in terrestrial habitats than in aquatic habitats, and terrestrial communities experience more severe homogenization in higher latitudinal regions. These findings are crucial for predicting the dynamics of invasive plant communities under rapid global change.

## Introduction

Under rapid global change, alien plant invasions increasingly cause serious threats to biodiversity, community stability and ecosystem functioning (Reaser et al., 2020; Peng et al., 2022). Many invasive plants significantly change the structure of native communities through interspecific competition and accelerate community homogenization (Vítková et al., 2020; Wang et al., 2021a). Lots of studies have showed that plant invasions greatly reduce the biomass, coverage, evenness, richness and diversity of native plant species (Flory and Clay, 2010; Yuguda et al., 2022), and plant invasions also could cause a significant increase in the litter biomass of invaded communities, which further benefits for excluding native plants (Gooden and French, 2014). Additionally, plant communities containing only native species have a higher invasibility than those that included alien species, and alien plants that have similar functional traits with natives are more easily to enter and dominate the native plant communities (Gooden and French, 2015; Li et al., 2015; Rijal et al., 2017). Moreover, environmental changes may affect the invasion process, e.g., seedling survival rate and drought tolerance of the invasive species *Pennisetum ciliare* are all increased by combinations of drought and warming, while this synergistic effect decreases leaf respiration of the native accompanying species *Heteropogon contortus* and causes substantial carbon loss, which accelerates native plant death (Ravi et al., 2022).

Latitudinal variations cause hydrothermal fluctuations, which could significantly affect the performance of invasive plants (Liu et al., 2022). The invaders usually have more active responses to latitudinal variations than their native congeners. E.g., the flowering period, biomass, individual height, seed size, leaf length-width ratio, water-use efficiency, seed abscisic acid and aliphatic acid contents of many invasive plants all increase with increasing latitude (Li et al., 2016; Zhou et al., 2021; Liu et al., 2022), and they also increase the reproductive allocation at higher latitudes by advancing the flowering onset (Helsen et al., 2020), which all benefits the expansion of invasion scopes. The territory of China spans approximately 50 degrees of latitude, and the variable climatic conditions along the latitudinal gradient provide conditions for plant invasions. Although the richness of invasive plants is high in the low latitudes of southern China, most have a lower noxious risk, while the invaders in the high latitudes of northern China have low richness but pose a higher risk (Feng and Zhu, 2010). In addition, global warming will accelerate the spread of these invasive plants originating from South America and Mexico to the high latitudinal regions of China (Feng et al., 2011). Thus, exploring the dynamics of invasive plant communities along latitudinal gradients is critical for maintaining native community stability and predicting plant invasion situations under global change.

Beta (β) diversity represents the differentiation of species composition and turnover between communities across environmental gradients. Compared with alpha (α) diversity, it is more conducive to understand community construction process and optimize biodiversity protection measurements at the larger biogeography scale (Cao et al., 2021; Yu et al., 2021). Community similarity is usually negatively related to β diversity; higher similarity indicates that communities tend to be stable, while higher β diversity shows faster species turnover (Chao and Chiu, 2016; Qian et al., 2022). Neutral theory proposes that community similarity decreases with increasing spatial distances, while β diversity is formed by the combined effect of species dispersal and ecological drift, which are also affected by climatic factors and soil types; in fact, these various environmental factors are comprehensively reflected by variations in latitude (Martin and Wilsey, 2015; Ricotta et al., 2017; Godsoe et al., 2022; Peng et al., 2022). Qian (2009) found that the β diversity of seed plants and fern plants in North America shows negative correlations with latitude, while Martin and Wilsey (2015) found that the community similarity of grasslands in Texas decreases with increasing latitude. However, latitudinal trends in the similarity and β diversity of plant community with single dominant invader are not well understood.

The alligator weed *Alternanthera philoxeroides* is a noxious invasive weed that is native to South America and has spread to a latitudinal gradient of 42°S to 37°N around the world (Wu et al., 2016). Because of its strong phenotypic plasticity, it has extensively invaded the aquatic and terrestrial habitats of nearly 20 provinces in China, causing a serious loss of native biodiversity (Wu et al., 2017a; Wu and Ding, 2019). Recently studies have shown that α diversity within the *A*. *philoxeroides* community decreases with increasing latitude in China (Wu et al., 2016). The chemical defence and growth rate of *A*. *philoxeroides* show opposite variations along a latitudinal gradient (Yang et al., 2021). Lu et al. (2013) found that climate warming facilitated faster *A*. *philoxeroides* expansion to higher latitudes than that of the biocontrol beetle *Agasicles hygrophila* and thus increased enemy release in those regions. Lu et al. (2016, 2018) also found nonparallel latitudinal patterns of rhizospheric pathogenic fungi and root-knot nematodes associated with *A*. *philoxeroides* and the native *A*. *sessilis*, which may enhance bioresistance to *A*. *philoxeroides* invasion at higher latitudes under climate change. However, differences among plant communities invaded by *A*. *philoxeroides* along a latitudinal gradient are unclear. In this study, we hypothesize that the structure, similarity and β diversity of the *A*. *philoxeroides* community may be significantly affected by latitude. Specifically, we addressed the following questions: (1) Are invasive dominance, species distribution, community similarity and β diversity in terrestrial and aquatic *A*. *philoxeroides* communities different? (2) Do these four parameters vary across a latitudinal gradient?

## Materials and methods

### Field survey and data collection

**Figure 1:**
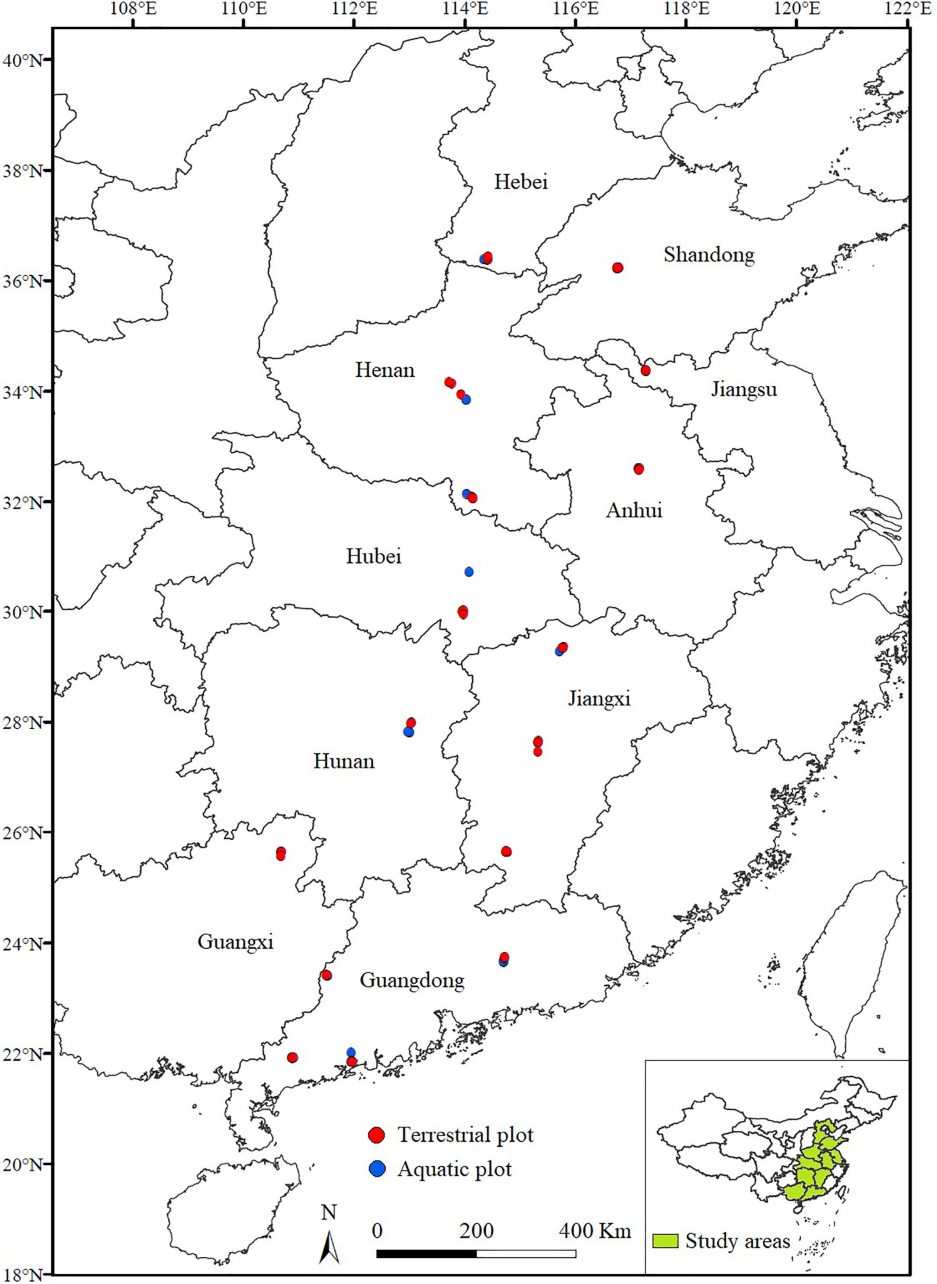
Sampling plot distributions of *A*. *philoxeroides* communities across amphibious habitats along a latitudinal gradient in China.

We conducted field surveys of *A*. *philoxeroides-*invaded communities during July and August of 2019-2020. Our study areas covered 18 cities of 10 provinces in China, which nearly contain the latitudinal bounds of present wild *A*. *philoxeroides* distributions in mainland China. We chose locations where *A*. *philoxeroides* covered an area of more than 100 m^2^ to establish plots. We selected the plots along eight latitudinal clusters (approximately 2° apart) from 21°N to 37°N in China (Clusters 1 to 8). In each latitudinal cluster, we selected five terrestrial plots (10×10 m for each) scattered in two or three sampling cities and selected five aquatic plots in the same latitudinal cluster close to terrestrial plots. We established 40 terrestrial and 40 aquatic plots, and plots with the same habitat type were at least 10 kilometers apart (as shown in **Figure 1**). The detailed geographic information of the plots is shown in the **Supplementary Table 1**.

We evenly set up three 10 m transects in each plot, and five quadrats (0.5×0.5 m) were uniformly spaced 2 m apart along each transect. According to the survey methods of Wu et al. (2016), we recorded the species name, abundance, individual height and species coverage of plants in each quadrat. We used the ‘Flora of China’ website (http://www.iplant.cn/frps) and the ‘Chinese Virtual Herbarium’ website (http://www.cvh.ac.cn) to identify unknown plant species. We also measured the longitude, latitude and elevation data of each plot using a handheld GPS receiver (Garmin GPSMAP 639csx, USA).

### Data analysis

We used the importance value (*IV*) to evaluate the dominance of plant species in each plot, and the *IV* was also used as matrix data in the numerical ordination of plant species distributions in this study. The calculation formula of *IV* in each plot was as follows (Wu et al., 2016; Wu et al., 2017a; Wu et al., 2017b):


IV=(relative abundance+relative height+relative coverage)/3

Where relative abundance, relative height and relative coverage refer to the percentages of each species abundances, height and coverage over the sum of all species total abundances, height and coverage within a plot, respectively.

The total *IV* was the sum of a plant species’ *IV* in all sampling plots.

We calculated the Jaccard index and Sorenson index using the following formulas, to evaluate the community similarity (Marzialetti et al., 2021):


$$C_{Jaccard}=j/(a+b-j)$$

$$C_{Sorenson}=2\;j/(a\;+\;b)$$

where a and b are the total number of plant species in communities A and B, respectively, and j is the number of plant species shared between these two communities.

β diversity represents the variation among communities in both species composition and abundance values, and we use the Bray−Curtis index and the β_sim_ index to evaluate β diversity with the following formulas (Chao and Chiu, 2016):


$$C_{Bray-Curtis}=2\;j_{N}/(N_{a}+\;N_{b})$$

$$C_{\beta sim}=min(b,c)/\left[a+min(b,c)\right]$$

where N_a_ and N_b_ are the total abundance values of all species in communities A and B, respectively; jN= ∑ min (jN_a_, jN_b_). a is the number of plant species shared between communities A and B, while b and c are the number of unique plant species in each community.

We conducted one-way analysis of variance (ANOVA) and multiple comparisons of Fisher’s protected least significant difference (LSD) (Subset for α = 0.05) by using SPSS 16.0 software (SPSS Inc., Chicago, USA) to examine the differences in *A*. *philoxeroides* IVs along the eight latitudinal clusters, and normality and homogeneity of our data were verified by using the homogeneity of variance test before ANOVA. We also examined the difference in *A*. *philoxeroides* IV, accompanying species richness, community similarity and β diversity between terrestrial and aquatic habitats by using the independent sample *t* test (Subset for α = 0.05) in SPSS 16.0.

We established the *IV* matrix (40×30) based on the main plants (top 30 plant species in total *IV*) in the terrestrial and aquatic habitats, respectively and established the latitude matrix (40×1 for terrestrial and 40×1 for aquatic). We then conducted detrended canonical correspondence analysis (DCCA) using Canoco 4.5 software (Microcomputer Power, Ithaca New York, USA) to explore the effect of latitudinal gradients on the plant species distributions in *A*. *philoxeroides-*invaded communities in heterogeneous habitats (Feurdean et al., 2012).

## Results

### Species dominance and composition along latitudinal gradients

**Figure 2:**
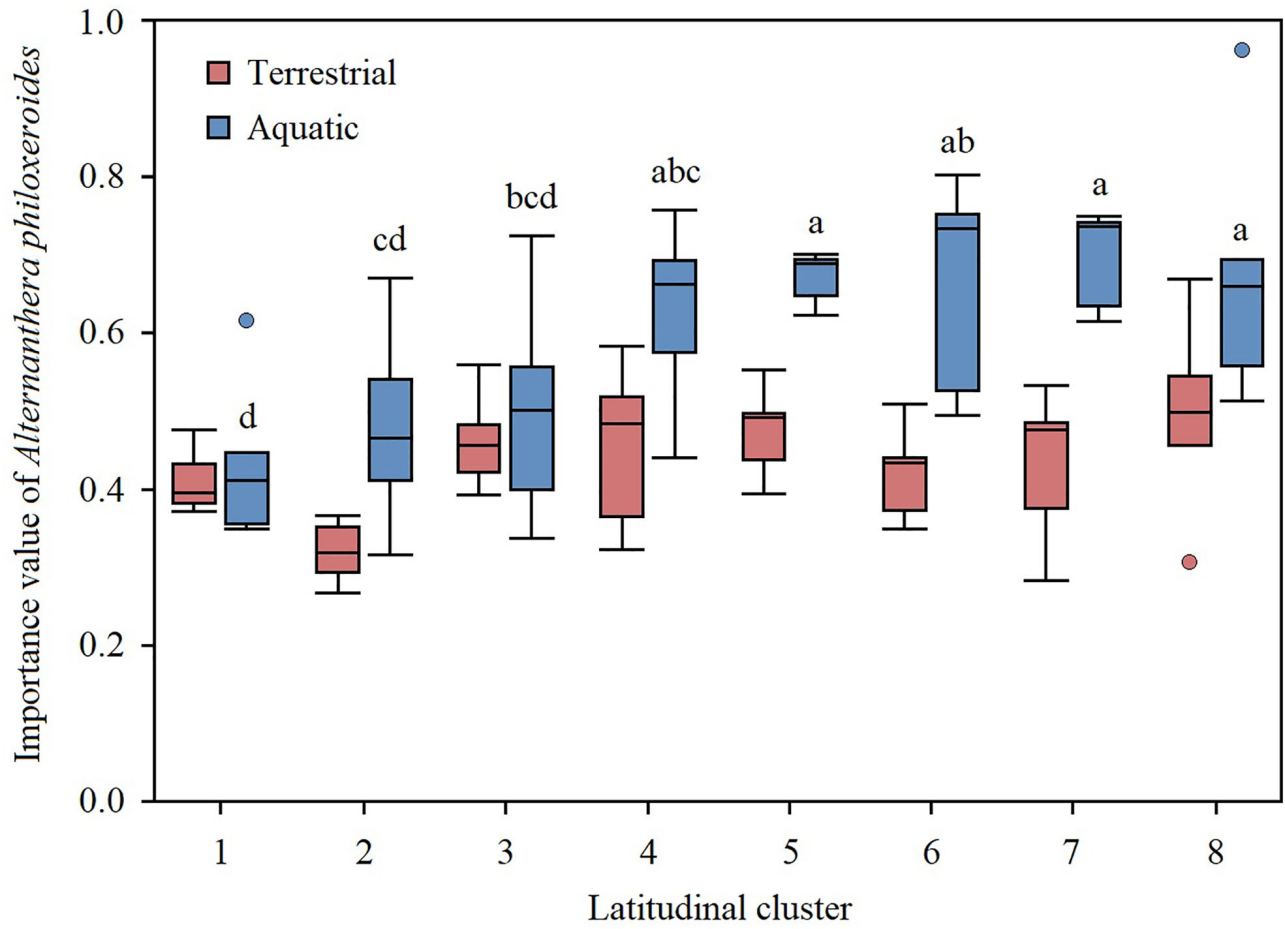
Variations in *A*. *philoxeroides* dominance in terrestrial and aquatic habitats among different latitudinal transects. The upper and lower bounds of the box represent the 75% and 25% percentiles, respectively; the line in the middle represents the mean and the whiskers extended to the 5% and 95% percentiles, respectively. Solid dots represent the outliers. The different letters indicate that the importance value has significant differences at the 0.05 level.

The mean *A*. *philoxeroides IV* of the terrestrial and aquatic plots was 0.434 and 0.594, respectively, and the *A*. *philoxeroides IV* in the aquatic habitats was extremely significantly higher than that in the terrestrial habitats (*t*=5.820, *P*<0.001). There was no significant difference in the *A*. *philoxeroides IV* in the terrestrial habitats among the eight latitudinal clusters, while there was an extremely significant difference in the aquatic habitats (F_7, 39 =_ 3.481, *P*=0.007) (**Figure 2**). Specifically, the aquatic *A*. *philoxeroides IV*s at higher latitudes (Clusters 7 and 8) were significantly higher than those at lower latitudes (Clusters 1 to 3) (**Figure 2**).

**Figure 3:**
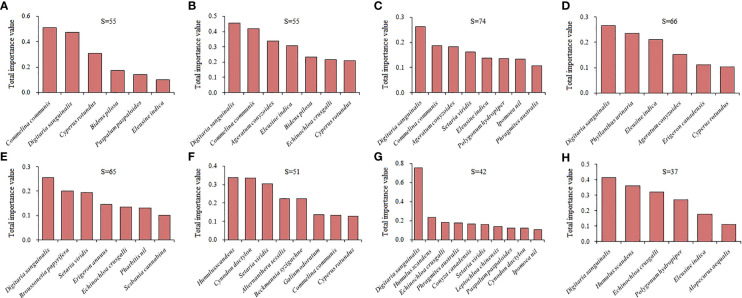
Variations in the dominant accompanying plant species of the *A*. *philoxeroides* communities among different latitudinal clusters in terrestrial habitats. S values represent the total number of accompanying plant species in the *A*. *philoxeroides-*invaded communities along each latitudinal cluster. **(A–H)** represent the 1 to 8 latitudinal clusters, respectively. The same is as follows.

**Figure 4:**
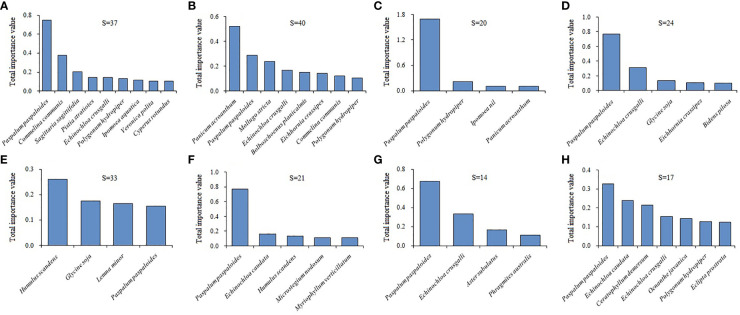
Variations in the dominant accompanying plant species of the *A*. *philoxeroides* communities among different latitudinal clusters in aquatic habitats. **(A–H)** represent the 1 to 8 latitudinal clusters, respectively.

We recorded 178 plant species belonging to 51 families and 149 genera in 40 terrestrial plots. Asteraceae had the most species (31 spp.), followed by Poaceae (21 spp.). We recorded 104 plant species belonging to 43 families and 90 genera in 40 aquatic plots, and Poaceae (16 spp.) and Asteraceae (11 spp.) had higher species richness. The mean accompanying species richness of eight latitudinal clusters in the terrestrial habitats was extremely significantly higher than that in the aquatic habitats (*t*=5.354, *P*<0.001), and the highest difference was in latitudinal Cluster 3 (21°N-37°N) (**Figures 3**, **4**).

In the terrestrial *A*. *philoxeroides* communities, the dominant accompanying species were *Digitaria sanguinalis*, *Commelina communis*, *Humulus scandens*, *Echinochloa crusgalli* and *Eleusine indica* (**Figure 3**). *D*. *sanguinalis* had the highest dominance in the most latitudinal clusters. The dominance of *C*. *communis*, *Cyperus rotundus* and *Bidens pilosa* decreased with increasing latitude. The dominance of *H*. *scandens* and *E*. *crusgalli* rapidly increased north of 31°N (latitudinal Cluster 6 to 8). In the aquatic communities, *Paspalum paspaloides*, *Polygonum hydropiper*, *Panicum acroanthum* and *C*. *communis* were the dominant accompanying species (**Figure 4**); among them, *P. paspaloides* had the highest dominance. The dominance of tall hygrophytes (e.g., *Oenanthe javanica*, *Phragmites australis*), as well as submerged plants (e.g., *Myriophyllum verticillatum*, *Ceratophyllum demersum*), increased at the higher latitudes.

### Species distribution along latitudinal gradients

**Figure 5:**
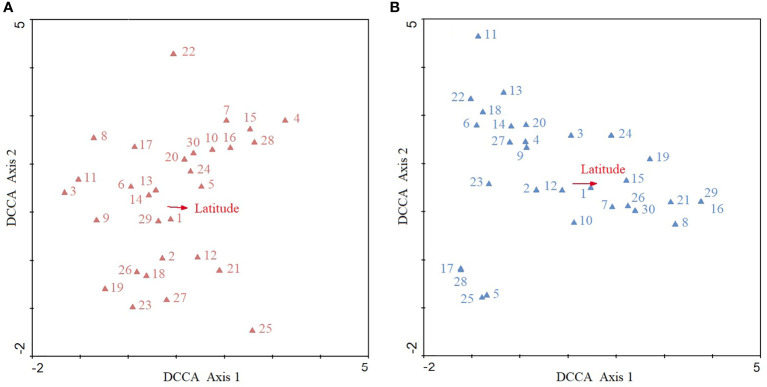
DCCA ordination diagrams of 30 dominant plant species of the *A*. *philoxeroides* communities in terrestrial **(A)** and aquatic **(B)** habitats along a latitudinal gradient. The triangle is the optimal distribution position of each plant species along the latitudinal gradient. The arrow indicates the direction of increase in latitude from the ordination centre. The vertical distance between the triangle and vector represents the correlation between the species distribution and latitude. Arabic numbers are codes of the dominant plant species (as **Table 1** shows for terrestrial and aquatic habitats).

Top 30 plant species in total *IV* in the amphibious habitats are shown in **Table 1**. The terrestrial communities had an obvious latitudinal trend of species distributions in the DCCA ordination diagram (**Figure 5A**). *C*. *communis* (3), *Ageratum conyzoides* (11), *C*. *rotundus* (8) and *B*. *pilosa* (9) are all distributed in the far left of the ordination diagram, showing that these plant species were intensively distributed in the lower latitudinal regions. Conversely, *H*. *scanden* (4) tended to be distributed in higher latitudinal regions. *A*. *philoxeroides* (1) had wide latitudinal adaptability, and it drastically competed with *Ludwigia prostrate* (29) as these two species located very closely. In addition, the distributions of *Oxalis corniculata* (22) and *Beckmannia syzigachne* (25) were weakly affected by latitude.

**Table 1:** The total importance values of dominant plant species in terrestrial and aquatic *Alternanthera philoxeroides* communities.

Terrestrial habitat			Aquatic habitat		
Code	Species name	Total *IV*	Code	Species name	Total *IV*
1	*Alternanthera philoxeroides*	17.362	1	*Alternanthera philoxeroides*	23.752
2	*Digitaria sanguinalis*	2.936	2	*Paspalum paspaloides*	5.427
3	*Commelina communis*	1.301	3	*Echinochloa crusgalli*	1.226
4	*Humulus scandens*	1.120	4	*Gnaphalium multiceps*	0.727
5	*Echinochloa crusgalli*	1.112	5	*Panicum acroanthum*	0.627
6	*Eleusine indica*	1.053	6	*Commelina communis*	0.534
7	*Setaria viridis*	0.972	7	*Humulus scandens*	0.444
8	*Cyperus rotundus*	0.901	8	*Echinochloa caudata*	0.403
9	*Bidens pilosa*	0.739	9	*Bidens pilosa*	0.366
10	*Cynodon dactylon*	0.695	10	*Glycine soja*	0.309
11	*Ageratum conyzoides*	0.680	11	*Mollugo stricta*	0.291
12	*Gnaphalium multiceps*	0.677	12	*Lemna minor*	0.284
13	*Pharbitis nil*	0.599	13	*Eichhirnia crasslpes*	0.253
14	*Paspalum paspaloides*	0.435	14	*Pharbitis nil*	0.232
15	*Erigeron canadensis*	0.425	15	*Eclipta prostrata*	0.217
16	*Phragmites australis*	0.359	16	*Ceratophyllum demersum*	0.215
17	*Artemisia argyi*	0.342	17	*Sagittaria trifolia* var. *sinensis*	0.201
18	*Alternanthera sessilis*	0.313	18	*Cyperus votundus*	0.198
19	*Cyperus votundus*	0.303	19	*Symphyotrichum subulatum*	0.197
20	*Broussonetia papyifera*	0.296	20	*Eleusine indica*	0.190
21	*Leptochloa chinensis*	0.284	21	*Phragmites australis*	0.187
22	*Oxalis corniculata*	0.250	22	*Alternanthera sessilis*	0.184
23	*Phyllanthus urinaria*	0.234	23	*Ipomoea aquatica*	0.169
24	*Erigeron annuus*	0.231	24	*Cyperus rotundus*	0.169
25	*Beckmannia syzigachne*	0.223	25	*Bolboschoenus planiculmis*	0.152
26	*Acalypha australis*	0.198	26	*Microstegium nodosum*	0.146
27	*Eclipta prostrata*	0.195	27	*Acorus calamus*	0.146
28	*Amaranthus lividu*	0.170	28	*Pistia stratiotes*	0.144
29	*Ludwigia prostrata*	0.168	29	*Oenanthe javanica*	0.143
30	*Galium odoratum*	0.137	30	*Typha orientalis*	0.141

The latitudinal trend of species distributions in aquatic habitats was more obvious (**Figure 5B**). *C*. *communis* (6), *A*. *sessilis* (22), *C*. *rotundus* (18) and *Ipomoea aquatica* (23) were distributed in lower latitudes, while *C*. *demersum* (16), *O*. *javanica* (29), *P*. *australis* (21) and *Echinochloa caudata* (8) tended to be distributed at higher latitudes. *A*. *philoxeroides* (1) have intense interspecific competition with *Lemna minor* (12). However, latitude weakly affected the distributions of *Mollugo stricta* (11), *Sagittaria trifolia* var. *sinensis* (17), *Pistia stratiotes* (28), *Bolboschoenus planicalmis* (25) and *P*. *acroanthum* (5).

### Community similarity and β diversity along latitudinal gradients

For community similarity, the Jaccard index (*t*=3.078, *P*=0.010) and Sorenson index (*t*=3.035, *P*=0.010) in the terrestrial communities were significantly higher than those in the aquatic communities. For β diversity, the Bray−Curtis index (*t*=4.002, *P*=0.006) and β_Sim_ index (*t*=2.302, *P*=0.040) in the aquatic communities were significantly higher than those in the terrestrial communities.

**Figure 6:**
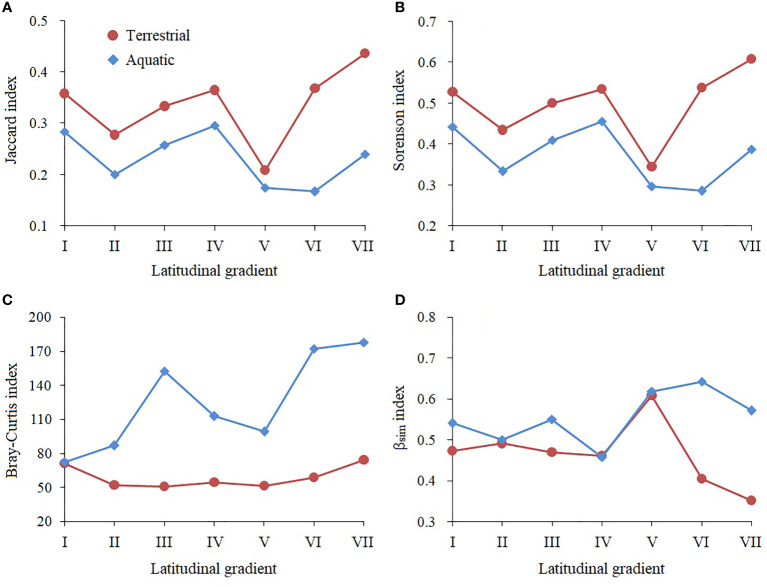
Variations in the β diversity index and community similarity index of the *A*. *philoxeroides* communities along the latitudinal gradient. Latitudinal gradients I to VII represent the pairwise comparison between the *A*. *philoxeroides* community across latitudinal Clusters 1 to 8. **(A, B)** represent the community similarity, **(C, D)** represent the β diversity.

The latitudinal variation in community similarity is shown as **Figures 6A, B**. With the increasing latitudinal gradient, terrestrial and aquatic community similarity showed similar repeated fluctuations. The terrestrial communities had the highest similarity at the higher latitudinal gradient (VII) and the lowest similarity at the middle latitudinal gradient (V). The aquatic communities had the highest similarity at the middle latitudinal gradient (IV) and the lowest similarity at the higher latitudinal gradient (VI).

The latitudinal variation in the β diversity between the terrestrial and aquatic communities showed great differences (**Figures 6C, D**). The terrestrial Bray−Curtis index was relatively stable with latitude, while the aquatic Bray−Curtis index was fluctuated, with a maximum value at a higher latitudinal gradient (VII) and a minimum value at a lower latitudinal gradient (I). The terrestrial β_sim_ index showed a rising first and then declining trend with latitude; it had a maximum value at the middle latitudinal gradient (V) and a minimum value at the higher latitudinal gradient (VII). However, the aquatic β_Sim_ index fluctuated greatly, with a maximum value at the higher latitudinal gradient (VI) and a minimum value at the middle latitudinal gradient (IV).

## Discussion

Habitat heterogeneity promotes niche differentiation, which provides more opportunities for plant species coexistence and affects the structure of an invaded community (Czarniecka-Wiera et al., 2020). In our study, the aquatic *A*. *philoxeroides IV* was significantly higher than the terrestrial *A*. *philoxeroides IV* along the whole latitudinal gradient, possibly because although *A*. *philoxeroides* invades both terrestrial and aquatic habitats in China, it prefers to invade aquatic ecosystems around the word (e.g., in the United States, New Zealand, and Australia), showing its original aquatic preference (You et al., 2018). In addition, the richness of terrestrial plants in the world is much higher than that of aquatic plants; therefore, the terrestrial bioresistance of a native community in terms of inhibiting an *A*. *philoxeroides* invasion may be higher than that of aquatic plants (You et al., 2016; Wu et al., 2017b), which further facilitates aquatic *A*. *philoxeroides* dominance and its significant latitudinal trend. In particular, the resource pulses caused by climate change in different latitudinal regions will also significantly change invaded community structures (Lu et al., 2018; Jentsch and White, 2019).

One of the important factors driving plant species coexistence in invaded communities is interspecific competition (Wang et al., 2021b). *D*. *sanguinalis*, *E*. *crusgalli* and *Setaria vulgaris* in terrestrial *A*. *philoxeroides* communities have rapid tillering ability and/or relatively higher individual height and can effectively resist *A*. *philoxeroides* invasion, and these plants could be considered biosubstitute species for invasion control (Wu et al., 2017a; Wu et al., 2017b; Yu et al., 2020). In addition, the *B*. *pilosa* in terrestrial communities possesses a larger seed yield and faster growth rate, combined with the capacity of uniparental reproduction, making it able to coexist with *A*. *philoxeroides* (Hao et al., 2018). The DCCA indicated that the proportion of cold-and drought-tolerant plant species rose with increasing latitude in the terrestrial communities. For example, native *H*. *scandens* at higher latitudes has superior stress resistance and vigorous twining stems allowing it to occupy spatial resources, and its strong allelopathy could also inhibit *A*. *philoxeroides* growth (Wang et al., 2021c). The dominant accompanying species in the aquatic habitats gradually changed from hygrophytes and floating plants to emerged and submerged plants with increasing latitude. For instance, *P*. *paspaloides* has strong photosynthetic carbon absorption and assimilation rates, allowing it to widely co-occur in aquatic invasive communities. *O*. *javanica*, *P*. *australis*, *M*. *verticillatum* and *C*. *demersum* have higher adaptability to water level fluctuations, which allows them to fully use heterogeneous light sources in aquatic ecosystems and coexist with invaders (Crisman et al., 2014; Saluja and Garg, 2017).

Plant invasions usually facilitate species turnover and the homogenization of flora, thus reducing β diversity (Yu et al., 2021; Petsch et al., 2022). In our study, the aquatic *A*. *philoxeroides* communities had lower similarity and higher β diversity than those of the terrestrial communities, which may have been related to the simpler community structure and the narrower species niche in the aquatic habitats that made them more vulnerable to external environmental fluctuations at large latitudinal scales; thus, they had higher rates of species turnover (Pennington et al., 2006; Wu et al., 2017b). In addition, comparing with the terrestrial habitats, aquatic habitats usually have the higher thermal stability, which makes aquatic plants have higher energy utilization rate than terrestrial plants, and thus benefits improving the specialization and species turnover rates of aquatic plants (Landeiro et al., 2014; Wu et al., 2017b).

Previous studies found that the α diversity of terrestrial *A*. *philoxeroides* communities was higher than that of aquatic communities, which create a higher bioresistance to terrestrial *A*. *philoxeroides* invasion and may reduce species turnover between invaders and native plants (Wu et al., 2017b). In fact, as a plant species, the dispersal of *A*. *philoxeroides* into terrestrial communities across a large latitudinal scale also directly increases community similarity by increasing the number of shared species (Ricotta et al., 2017). Our study emphasized that only the plant species with traits for resisting *A*. *philoxeroides* invasion could continuously occur in the invaded communities. Similar to Poaceae and Asteraceae, these highly invasion-tolerant functional groups occupy a large proportion of terrestrial accompanying species compositions in all latitudinal clusters. This may be because with an ongoing invasion, the phylogenetic relationships in the terrestrial *A*. *philoxeroides* community experience rapid convergent evolution, which increases community similarity, and a similar phenomenon has also been found in terrestrial invaded communities in India and North America (Dar and Reshi, 2015; Stotz et al., 2016; Petsch et al., 2022). Compared to aquatic communities, terrestrial *A*. *philoxeroides* communities experience stronger disturbances from human activities, climate change and herbivores, which may interrupt ongoing species turnover and thereby reduce β diversity (Lu et al., 2016; Ricotta et al., 2017; Xiao et al., 2020).

The latitudinal trend of community similarity and β diversity in terrestrial habitats may be associated with the growth-defence tradeoffs of invasive plants (Ulrich et al., 2014; Martin and Wilsey, 2015). As the variations of microclimate and herbivore pressure along latitudes, terrestrial *A*. *philoxeroides* synthesizes more secondary metabolites at mid-latitudes, for increasing the chemical defence, but relatively reduces its growth and dominance (Liu et al., 2021). This may provide more opportunities for native species establishment and turnover, thus decreasing community similarity in middle latitudes. However, *A*. *philoxeroides* spreads faster than its natural enemy *A. hygrophila* along latitudes with recent global warming, and this invader has shifted its defence strategy to a growth strategy at higher latitudes due to ‘enemy release’ (Lu et al., 2013). This scenario would generally increase the component proportion of *A*. *philoxeroides* in the invaded terrestrial community and increase community similarity at higher latitudes. These findings also indirectly support the latitudinal herbivory defence hypothesis (LHDH), which predicts that the herbivory damage and plant defence all increase toward lower latitudes (Johnson and Rasmann, 2011). In this study, the terrestrial Bray−Curtis index was not sensitive to latitudinal gradients, perhaps because this parameter was calculated based on abundance, and our survey was conducted in summer when terrestrial plants had higher species richness values; thus, the response of richness to latitude masks that of abundance. Field surveys in different seasons should be conducted in the future to more accurately evaluate the β diversity of invaded terrestrial communities.

In our study, aquatic *A*. *philoxeroides* had lower similarity but higher β diversity at higher latitudinal gradients, which was associated with the dispersal limitation of native aquatic plant species (Qian, 2009; Shi et al., 2021). Compared with the developed hydrographic networks of central and southern China, the number of wild aquatic habitats in the higher latitudinal regions of North China have sharply decreased (Liu et al., 2017), which has reduced the connectivity of the aquatic plant community at higher latitudes with that at middle and lower latitudes. This may cause greater geographical isolation and benefit aquatic species specialization at higher latitudes and decrease the community similarity in those regions (Wu and Ding, 2019). For instance, in comparison to other species, the emerging dominant accompanying species *C*. *demersum* in aquatic habitats in higher latitudinal clusters has superior asexual reproduction and high assimilation efficiency of nitrogen (Xiong et al., 2010), and these advantages in competition may lead to higher species turnover.

In conclusion, we found significant differences in the structure, similarity and β diversity of *A*. *philoxeroides* communities in heterogeneous habitats, as well as their latitudinal trends, which were quite different from the latitudinal α-diversity gradient (LDG) rule of the *A*. *philoxeroides* community in our previous studies (Wu et al., 2016; Wu et al., 2017b). These results indicate that the terrestrial and aquatic *A*. *philoxeroides* communities had higher species turnover rates at middle and high latitudes, respectively, and native plants in these regions have potential substitutions for *A*. *philoxeroides* invasions. However, a rainfall belt moving northwards in China would further improve the connectivity of aquatic *A*. *philoxeroides* invasions, which would increase community similarity at higher latitudes (Wu and Ding, 2020), while climate warming aggravates the nontarget effects of terrestrial *A*. *philoxeroides* invasion control at middle latitudes and may thus decrease terrestrial β diversity (Lu et al., 2015; Liu et al., 2021). We should intensify the invasion assessment of *A*. *philoxeroides* at middle and high latitudes to prevent community homogenization in those regions. Our study explored the differences among *A*. *philoxeroides*-invaded communities among all latitudinal bounds of its present distributions on mainland China, and these findings could provide important implications for better understanding the construction process in invaded communities and predicting plant invasions under global change.

## Data availability statement

The original contributions presented in the study are included in the article/**Supplementary Material**. Further inquiries can be directed to the corresponding authors.

## Author contributions

HW and BR designed the experiments. HW and SD performed the experiments and analyzed the data. HW, SD and BR wrote the manuscript. All authors contributed to the article and approved the submitted version.

## Funding

This study was funded by the National Natural Science Foundation of China (31800460) and the Nanhu Scholars Program for Young Scholars of Xinyang Normal University (XYNU).

## Acknowledgments

We would like to thank Yanyan Wang, Li Wang, Junhui Jiang, Tiantian Zhang, Qiubo Ji, Nannan Xiao, Wenhao Wang, Shaoqi Jia, Junda Wang for their help in field surveys and laboratory analysis. We are grateful for comments by the editor and reviewers that improved this manuscript. We are also grateful to Springer Nature Author Services for language editing.

## Conflict of interest

The authors declare that the research was conducted in the absence of any commercial or financial relationships that could be construed as a potential conflict of interest.

## Publisher’s note

All claims expressed in this article are solely those of the authors and do not necessarily represent those of their affiliated organizations, or those of the publisher, the editors and the reviewers. Any product that may be evaluated in this article, or claim that may be made by its manufacturer, is not guaranteed or endorsed by the publisher.
