# Supplementary material for: Latitudinal trends in the structure, similarity and beta diversity of plant communities invaded by Alternanthera philoxeroides in heterogeneous habitats
Source: Front Plant Sci. 2022 Oct 6;13:1021337. doi: 10.3389/fpls.2022.1021337 (PMC9583019; doi:10.3389/fpls.2022.1021337)
Supplement: Supplementary file 1 [file Table_1.docx]

**Supplementary Table 1.** Location distributions of terrestrial and aquatic sampling plots invaded by *Alternanthera philoxeroides*

| Plot No. | Habitat type | Latitude / °N | Longitude / °E | Elevation / m | Plot No. | Habitat type | Latitude / °N | Longitude / °E | Elevation / m |
| --- | --- | --- | --- | --- | --- | --- | --- | --- | --- |
| 1 | Terrestrial | 21.905 | 110.876 | 4 | 41 | Aquatic | 21.911 | 110.864 | 11 |
| 2 | Terrestrial | 21.912 | 110.864 | 45 | 42 | Aquatic | 21.916 | 110.896 | 41 |
| 3 | Terrestrial | 21.919 | 110.885 | 43 | 43 | Aquatic | 21.842 | 111.963 | 17 |
| 4 | Terrestrial | 21.846 | 111.956 | 9 | 44 | Aquatic | 21.834 | 111.937 | 4 |
| 5 | Terrestrial | 21.834 | 111.957 | 17 | 45 | Aquatic | 21.997 | 111.939 | 9 |
| 6 | Terrestrial | 23.405 | 111.500 | 50 | 46 | Aquatic | 23.399 | 111.515 | 15 |
| 7 | Terrestrial | 23.403 | 111.500 | 30 | 47 | Aquatic | 23.648 | 114.700 | 40 |
| 8 | Terrestrial | 23.407 | 111.492 | 25 | 48 | Aquatic | 23.656 | 114.696 | 47 |
| 9 | Terrestrial | 23.724 | 114.713 | 39 | 49 | Aquatic | 23.658 | 114.694 | 46 |
| 10 | Terrestrial | 23.723 | 114.719 | 38 | 50 | Aquatic | 23.709 | 114.703 | 38 |
| 11 | Terrestrial | 25.639 | 110.677 | 223 | 51 | Aquatic | 25.641 | 110.676 | 221 |
| 12 | Terrestrial | 25.562 | 110.668 | 211 | 52 | Aquatic | 25.633 | 110.669 | 218 |
| 13 | Terrestrial | 25.635 | 114.770 | 136 | 53 | Aquatic | 25.633 | 110.669 | 215 |
| 14 | Terrestrial | 25.636 | 114.741 | 146 | 54 | Aquatic | 25.644 | 114.744 | 115 |
| 15 | Terrestrial | 25.643 | 114.743 | 130 | 55 | Aquatic | 25.642 | 114.743 | 136 |
| 16 | Terrestrial | 27.978 | 113.036 | 54 | 56 | Aquatic | 27.801 | 112.991 | 33 |
| 17 | Terrestrial | 27.965 | 113.016 | 48 | 57 | Aquatic | 27.802 | 112.991 | 40 |
| 18 | Terrestrial | 27.444 | 115.322 | 37 | 58 | Aquatic | 27.811 | 112.966 | 44 |
| 19 | Terrestrial | 27.638 | 115.329 | 70 | 59 | Aquatic | 27.616 | 115.319 | 38 |
| 20 | Terrestrial | 27.616 | 115.319 | 41 | 60 | Aquatic | 27.617 | 115.319 | 41 |
| 21 | Terrestrial | 29.991 | 113.945 | 29 | 61 | Aquatic | 30.719 | 114.072 | 25 |
| 22 | Terrestrial | 30.004 | 113.971 | 27 | 62 | Aquatic | 30.004 | 113.971 | 25 |
| 23 | Terrestrial | 29.941 | 113.973 | 32 | 63 | Aquatic | 29.330 | 115.765 | 12 |
| 24 | Terrestrial | 29.346 | 115.785 | 27 | 64 | Aquatic | 29.329 | 115.765 | 23 |
| 25 | Terrestrial | 29.329 | 115.765 | 18 | 65 | Aquatic | 29.264 | 115.708 | 40 |
| 26 | Terrestrial | 32.074 | 114.116 | 70 | 66 | Aquatic | 32.131 | 114.031 | 64 |
| 27 | Terrestrial | 32.046 | 114.146 | 75 | 67 | Aquatic | 32.061 | 114.129 | 73 |
| 28 | Terrestrial | 32.581 | 117.142 | 29 | 68 | Aquatic | 32.052 | 114.142 | 77 |
| 29 | Terrestrial | 32.585 | 117.158 | 25 | 69 | Aquatic | 32.593 | 117.134 | 33 |
| 30 | Terrestrial | 32.559 | 117.144 | 20 | 70 | Aquatic | 32.584 | 117.135 | 24 |
| 31 | Terrestrial | 33.927 | 113.922 | 72 | 71 | Aquatic | 33.837 | 114.017 | 49 |
| 32 | Terrestrial | 34.130 | 113.759 | 91 | 72 | Aquatic | 33.831 | 114.023 | 52 |
| 33 | Terrestrial | 34.148 | 113.713 | 83 | 73 | Aquatic | 34.370 | 117.268 | 30 |
| 34 | Terrestrial | 34.339 | 117.268 | 15 | 74 | Aquatic | 34.363 | 117.267 | 22 |
| 35 | Terrestrial | 34.368 | 117.267 | 27 | 75 | Aquatic | 34.370 | 117.259 | 36 |
| 36 | Terrestrial | 36.370 | 114.411 | 57 | 76 | Aquatic | 36.383 | 114.415 | 70 |
| 37 | Terrestrial | 36.422 | 114.418 | 65 | 77 | Aquatic | 36.369 | 114.402 | 70 |
| 38 | Terrestrial | 36.222 | 116.782 | 105 | 78 | Aquatic | 36.373 | 114.338 | 65 |
| 39 | Terrestrial | 36.217 | 116.763 | 96 | 79 | Aquatic | 36.208 | 116.742 | 92 |
| 40 | Terrestrial | 36.218 | 116.754 | 103 | 80 | Aquatic | 36.218 | 116.754 | 98 |
